# Evaluation of Characteristics Associated with Self-Identified Cat or Dog Preference in Pet Owners and Correlation of Preference with Pet Interactions and Care: An Exploratory Study

**DOI:** 10.3390/ani14172534

**Published:** 2024-08-31

**Authors:** Andrea Y. Tu, Cary Michele Springer, Julia D. Albright

**Affiliations:** 1 Department of Veterinary Behavior Medicine, Heart of Chelsea Veterinary Group, 257 West 18th Street, New York, NY 10011, USA; 2 Department of Small Animal Clinical Sciences, College of Veterinary Medicine, University of Tennessee, C247 Veterinary Medical Center, Knoxville, TN 37996, USAjalbrig1@utk.edu (J.D.A.)

**Keywords:** human–animal bond, pet preference, cat people, dog people, pet-owner interactions, pet food

## Abstract

**Simple Summary:**

There is evidence that cat and dog preferences are correlated with human personality traits, but little information exists on how these preferences develop and change over time and if preference impacts pet care, particularly of the “less preferred” species in a multispecies household. By using an internet survey, we found some differences in demographics and exposure to cats or dogs when young and those who prefer cats or dogs as adults. Species preference for dogs remained consistent from youth to adulthood, but for those that changed preference, we found that lack of childhood exposure impacted the formation of a cat-species preference but not of a dog-species preference. We also found that pet owners spent more time with their preferred species, cat people were more likely to feed their cats a prescription diet, and dog people were more likely to feed both their cats and dogs raw diets. Species preferences may result in preferential treatment of the owner’s preferred species and identified a potential risk for welfare concerns in multispecies households. More studies are needed to further examine the development and impact of species preferences.

**Abstract:**

Dog and cat preference has been associated with a few factors, like owner personality traits, but data regarding other aspects of preference ontogeny and the impact of preferences on pet wellbeing have yet to be examined. In this exploratory study, several of these characteristics, such as exposure to pets when young and as adults and current pet interactions and diet were analyzed from internet survey data. We found that more people identified as dog people (63.3%) versus cat people (36.7%) and preference for dogs remained consistent from childhood to adulthood compared with cats. In individuals who changed species preference, a lack of childhood exposure to cats (47.2%) was significantly associated with the group that changed preferences from dogs to cats from childhood to adulthood, compared with dog ownership as a child in the group that changed preferences from cats to dogs (24.4%). The number of cats and dogs in the home directly correlated with species preference (*p* < 0.001). Dwelling location was also significantly associated with species preference, with cat people being more likely to live in an urban area and dog people in a rural area (*p* = 0.002). More time was spent in both active and passive interactions with pets of the preferred species. Cats owned by cat people were more likely to be fed prescription diets compared with cats owned by dog people (*p* < 0.001). Interestingly, dog people were more likely to feed both their cats (*p* = 0.012) and dogs (*p* < 0.001) a raw diet compared with cat people. Additional research is needed to understand the development and impact of owner species preferences on pets to identify risks of suboptimal wellbeing.

## 1. Introduction

Cats and dogs are the most common pets in the United States, with an estimated 45% and 26% of households having at least one canine and feline companion, respectively. This equals about 89 million dogs in 62 million households and 37 million households shared with 62 million cats [1]. These statistics are consistent with the 2021–2022 American Pet Products Association National Pet Owner’s Survey, which further found that 21% or 27.2 million households own both a cat and a dog [2]. Dogs are not only the most popular pet, but the number of US households reportedly owning a dog has steadily increased over time, whereas the number of US households with cats has fluctuated [1]. The many differences in genetics, social behavior, and human-owner personality traits between cats and dogs may account for their disproportionate popularity among US residents.

Dogs (*Canis familiaris*) are considered the first domestic species. Molecular genetic data suggest that they began to emerge as a unique species from the gray wolf (*Canis lupis*) approximately 15,000–40,000 years before present (BP) during the transition from hunter–gather to agrarian human cultures [3,4,5]. Cats (*Felis catus*), the only modern domestic species to have evolved from a solitary ancestor (African wildcat *Felis lybica*), arose much more recently (approximately 10,000 BP) coinciding with more sedentary human societies [6]. The domestication of ancestral species began as commensal relationships, in which the animal was provided with consistent resources and, in return, served as sentinels or aided humans in hunting [7,8]. However, the great success of dogs and cats across human cultures cannot solely be attributed to their utilitarian nature. Cats, for example, are probably less efficient vermin exterminators than other species, such as the weasel [9]. It is hypothesized that humans were at least in part motivated to further taming proto-cats and -dogs, which was undoubtedly dangerous and required resource sharing, due to the more pronounced neotenous morphologic traits (e.g., large eyes and small mouth) in the species that strongly elicit caregiving tendencies [9,10]. Modern psychophysiological studies have found that similar attachment neurohormones (e.g., oxytocin) are involved in both mother–infant and human–dog interactions. Reciprocal oxytocin release can also be detected in the dog during affiliate interactions with the owner [11,12,13,14], but this has yet to be assessed in the cat–human relationship.

Although humans form bonds with both cats and dogs, the dog may have an advantage. The pre-existing social nature of the wolf ancestors and intense human selective pressure for distinct behavioral and physical traits have produced highly developed social cognition in the domestic dog, including sophisticated intraspecific communication abilities [15,16,17]. The domestic cat has not faced the same selective breeding pressure throughout most of its history, resulting in behavior and physiology that closely resembles their African wild cat ancestors [18]. However, domestication has provided the possibility for social behavior in cats [19]. The domestic cat has evolved human-specific communication, but research suggests that these behaviors are not as easily recognized by humans [20].

Cats can also form strong attachments to people [21]. Unlike “Man’s Best Friend,” the latency for cats to form social bonds with humans can be much longer, often requiring prolonged exposure and/or human interaction from an early age before displaying social interactions with humans [19,22]. One aspect underlying the delay in bonding with humans is the strong fight or flight threat response in cats. While both dogs and cats belong to the order Carnivora and are considered hunters, cats are also subject to predation by larger animals, and are more likely to default to prey-like escape, avoidance, or agonistic behaviors (“fight or flight”) in the presence of possible threats, including humans.

Intrinsic differences between feline and canine sociality and the difficulty for humans to interpret feline behavior might account for the variation in perceptions and attraction to domestic cats and dogs as companion animals (i.e., “cat people” and “dog people”). For example, it is common for cats to be thought of as independent and enigmatic, whereas dogs are found to be energetic and loyal. Americans tend to attribute masculine qualities to dogs and dog people, and feminine qualities to cats and cat people [23,24]. By using the Big Five personality dimensions—openness, conscientiousness, extraversion, agreeableness, and neuroticism—to assess correlations to species preferences, Gosling et al. [25] found that when compared with cat people, dog people scored higher on extraversion, conscientiousness, and agreeableness and lower on neuroticism and openness. Based on the personality inventory scales as defined by the Edwards Personal Preference Schedule (EPPS) [26], Perrine and Osbourne did not find a significant difference in athleticism nor dominance (defined by the EPPS as a need to be a leader and influence others) between cat and dog people but did find that dog people rated themselves as more independent than cat people [24].

Despite the breadth of studies on human personality traits and the correlation to species preferences, existing studies are inconclusive as to how self-identified cat and dog preferences are formed. While Kidd and Kidd [27] found that in general, people tended to prefer the type of pet they grew up with, other studies showed no relationship between species preferences with past and present dog or cat ownership [24]. However, the quality of past pet ownership experience could impact self-identified species preferences, in that cat people had more positive past experiences with cats than dog people, and dog people had more positive past experiences with dogs than cat people [24].

There are no studies examining whether cat people and dog people changed their species preference from youth to adulthood and factors that may impact that change in species preference. Moreover, few have looked at how “cat people” and “dog people” qualifiers impact the husbandry and the human–animal bond with different species. As many studies have shown a correlation to a strong human–animal bond and positive companion animal welfare [28,29,30,31], individual species preferences may alter the human–animal bond and subsequently impact the care and welfare of the preferred and less preferred species in a multispecies household. The aim of this study was to explore in a population of pet owners how self-identified labels of cat person and dog person may be associated with basic childhood exposure and how this self-identified cat or dog person label may relate to the care or environment of their pets. We proposed that due to the relative ease in which dogs form social bonds with humans, more individuals would learn to appreciate dogs even if they did not have one as a pet, ultimately resulting in more individuals identifying as dog people. Moreover, individuals would identify as a cat person and show a preference for cats following exposure to a cat in a home environment. We further proposed that in a multispecies household of both cats and dogs, the species that the participant prefers would receive additional positive contact and financial resources spent on pet care products (e.g., specialized pet food).

## 2. Materials and Methods

### 2.1. Study Design

A survey was designed to gather data investigating pet owners’ identification of their preferences for dogs or cats and how this preference might correlate with childhood exposure to cats or dogs, population density of current home location, amount of active and passive interaction pet owners engaged in with each pet species, and the type of diet they fed their pets. The questions analyzed are part of a larger survey looking at the interactions of people, cats, and dogs living in the same household. The survey was conducted using survey software (Qualtrics^®^, Version July 2023) and was available for two weeks in 2023 to residents of the United States over the age of 18 years who lived in a home with at least one pet at the time of the survey. Before answering the survey questions, participants were provided with brief instructions on answering the survey, purpose of the study, clarification that all responses were anonymous, and that they were eligible to withdraw from the study at any time before submission. As no personal identifiers were requested, the introductory material explained that withdrawal after submission was not possible. After review by the University of Tennessee Institutional Review Board, the study was granted exemption.

The link to the survey was distributed to several veterinary clinic reception areas and online by using social media. The questionnaire comprised of 43 multiple-choice and free-response questions aimed to collect demographic data (number cats and dogs in the home and urban–suburban–rural dwelling area), perception of self as a dog person or cat person currently and when younger (under the age of 18 years old), and presence of cats or dogs in the home when under the age of 18 years old and currently as adults.

Participants were also asked about the type of diet fed to cats and dogs in the home currently and to estimate how much time they spent in active play and passive interactions with their pets. For cats, active play was defined as how many minutes in a 24-h day the individual completing the survey engaged in activities such as playing fetch, using a fishing rod toy, etc. For dogs, active play was defined as how many minutes in a 24-h day the individual completing the survey engaged in activities such as playing fetch, tug, wrestling, etc., but participants were instructed to exclude time spent on walks and runs unless they played these games during the outing. While walks may contribute to a large time commitment to dog ownership, there is no equivalent measure for cat ownership. Moreover, Gunter et al. [32] found that the activity of walking dogs is not a good determinant of the strength of the bond between a dog and their owner, and that the quality of the time shared with one’s pet is more impactful on the human–animal bond [32]. Additionally, Scheibeck et al. [33] found that back yards are a confounding factor, as dog owners with a back yard walked their dogs less than those who did not have access to such a space. Due to these factors, our study excluded times spent on walks from our active interaction assessment. For active play in both cats and dogs, survey participants were asked to select from the following options: none; less than 15 min per day; 15–30 min per day; 30–45 min per day; 45–60 min per day; 60–90 min per day; 90–120 min per day; and more than 120 min per day. Similarly, participants were queried about the time they spend engaging in passive interactions with their pets. For both cats and dogs, passive interaction was defined as how many hours in a 24-h day the individual completing the survey spent in a passive state near their cats or dogs, defined as the pet is sitting/lying on or within a few feet of a human, but not actively playing with the human. Options included either: none; less than 1 h per day; 1–2 h per day; 3–5 h per day; 6–8 h per day; and more than 8 h per day. The questions that were included in this paper’s analysis are included in the Supplementary Materials (Supplementary Table S1).

### 2.2. Participants

A total of 914 surveys were completed. After excluding ineligible participants, including those that did not reside in the United States, were under 18 years of age, did not currently own either a cat or a dog, or did not complete the survey, there were 701 valid surveys.

### 2.3. Data Analysis

Descriptive statistics were determined for participants’ reported household demographics, including the number of cats and dogs in the household and urban–suburban–rural dwelling type; perceptions of cat or dog preference (“cat person” or “dog person” without option to choose both or neither species) both at the time of survey (adult) and when young (under 18 years of age); species of pets in the house both when young and currently; the amount of time spent in active play with pets and the amount of time spent in a passive state with pets; and the type of diet fed to pets.

During data analysis of active play time, the ranges were collapsed into the following groups: “Less than 15 min”, “15–45 min”, “45–90 min”, and “More than 90 min”. Similarly, the selections for passive interaction were grouped as follows: “Less than 3 h”, “3–5 h”, “6–8 h”, and “More than 8 h”.

Diet options to select from included a commercial prescription diet prescribed by the pet’s veterinarian for a medical condition; a commercial diet from the pet store or a special pet food subscription service; a commercial diet from a grocery store/supermarket/warehouse store; a homecooked diet; a raw diet; or other. Participants were asked to select all that applied for the cats and the dogs in the household.

Pearson chi-squared tests were performed to determine the differences between cat and dog people in types of pets currently owned, the dwelling location, pet preference when young, the amount of time spent in active play and passive interactions with cats and dogs in the home, and the type of diet fed to pets in the household. Mann–Whitney U tests were used to compare cat and dog people with regards to the median number of cats and dogs in the household. Pearson chi-squared tests were also used to examine how changes in pet preferences were related to exposure to cats and/or dogs when young. Statistical analyses were performed by using IBM SPSS Statistics 29.0 (IBM Corp., Armonk, NY, USA), with *p* < 0.05 being considered significant.

## 3. Results

### 3.1. Household Demographics

The final data set included 80 (11.4%) households currently owning cats only, 126 (18.0%) households that currently own dogs only, and 495 (70.6%) households that were multispecies and currently owned both cats and dogs. The median number of cats in cat-only households was 2 (range 1 to 8) and the median number of dogs in dog-only households was also 2 (range of 1 to 6). In multispecies pet households with both cats and dogs, there was a median of 2 cats (range 1 to 11) and 2 dogs (range of 1 to 8 dogs). The number of cats owned in a cat-only household versus a multispecies household did not significantly differ (*n* = 575, Z = −0.27, *p* = 0.787). The same was also true for dogs (*n* = 621, Z = −0.19, *p* = 0.849).

### 3.2. Species Preferences and Household Demographics

Of our 701 subjects, 257 (36.7%) self-identified as cat people, and 444 (63.3%) self-identified as dog people. Cat people owned significantly more cats (*n* = 701, Z = −9.92, *p* < 0.001), and dog people owned more dogs (*n* = 701, Z = −10.573, *p* < 0.001), with cat people owning a median of 2 cats and 1 dog, whereas dog people had a median of 1 cat and 2 dogs.

When further evaluating the number of pets in the home based on species preferences and type of households, cat people were more likely to have cat-only households (29.6%) and dog people were more likely to have dog-only households (27.9%) (*n*= 701, χ^2^ =184.06, df = 2, *p* < 0.001). For the multispecies households that had both cats and dogs, species preferences were split, and cat people (69.6%) and dog people (71.2%) were equally likely to maintain multispecies households (Table 1).

Pet-species preference significantly differed by population density of the current location (*n* = 701, χ^2^ =12.36, df = 2, *p* = 0.002) in that cat people were more likely to live in an urban area, and dog people were more likely to live in a rural area, with suburban areas having an equal distribution of cat people and dog people. In our sample population of 257 cat people, 43 (16.7%) reported living in a rural area, 155 (60.3%) in a suburban area, and 59 (23.0%) in an urban area. For the 444 dog people respondents, 116 (26.1%) lived in a rural area, 262 (59.0%) in a suburban area, and 66 (14.9%) in an urban area.

### 3.3. Changes in Species Preferences from Childhood to Adulthood

To determine how species preferences may have developed over time, we queried the species preference participants recalled having when young (less than 18 years of age). Out of the total 701 subjects, 225 (32.1%) and 476 (67.9%) self-identified as cat and dog people when young, respectively. Of the 257 respondents who currently self-identified as cat people, 140 (54.5%) considered themselves cat people when young and 117 (45.5%) were dog people when young. Of the 444 respondents who currently identify themselves as dog people, 359 (80.9%) were dog people when young and only 85 (19.1%) were cat people when young (Table 2). Individuals who currently identified as dog people were significantly more likely to have been a dog person from youth, but of those individuals that currently identified as a cat people had almost a 50% chance that they were either a dog person or a cat person growing up (*n* = 701, χ^2^ =93.23, df = 1, *p* < 0.001).

To better assess how childhood exposure to pets may have influenced the change in species preference, respondents were coded into four change groups based on their childhood and current pet preferences (e.g., cat preference when young changed to dog preference as adult is Cat–Dog). A total of 140 (20.0%) self-identified as cat people when young and continued to identify as cat people as adults (“Cat–Cat”), while 117 (16.7%) people who preferred dogs when young changed to a cat preference as adults (Dog–Cat). A total of 359 (51.2%) respondents considered themselves dog people when young and continue to keep this preference (Dog–Dog), while 85 (12.1%) of the respondents switched from a cat to dog preference as adults (Cat–Dog).

For those that changed preferences between childhood and adulthood, there was a significant difference in the species of childhood pets owned (*n* = 188, χ^2^ = 58.09, df = 2, *p* < 0.001). Individuals who changed from preferring cats to preferring dogs (Cat–Dog) were significantly more likely to have owned a dog as a child (Table 3). The majority of individuals in the Cat–Dog group grew up in a household with dogs (multispecies 74.4% + dog only 1.2% = 75.6%) compared with 24.4% who grew up without dogs. For those changing from a dog to cat preference (Dog–Cat), exposure to cats as a child did not greatly differ: specifically, 52.8% grew up in a household with cats (inclusive of both multispecies and cat-only households), while 47.2% grew up without exposure to cats.

Additionally, when we evaluated the species of pets owned currently, we found that the majority of those that changed preferences were more likely to have both cats and dogs. A total of 71.3% had a multispecies household compared with 27.7% having only a pet of their current preference, and there was no significant difference between Cat–Dog and Dog–Cat people (*n* = 188, χ^2^ = 0.698, df = 2, *p* = 0.705) (Table 4).

### 3.4. Species Preferences and Amount of Active and Passive Interaction with Pets and Diets Fed to Pets

Results comparing interactions and diet choices based on species preferences were split into households with cats (comprising both cat-only and multispecies households) and dogs (inclusive of both dog-only and multispecies households).

#### 3.4.1. Species Preferences and the Impact on Active and Passive Interactions in Cat-Owning Households

In cat-owning households, individuals who self-identified as cat people spent significantly more time engaging in active play (*n* = 575, χ^2^ = 11.78, df = 3, *p* = 0.008) and in passive interaction (*n* = 569, χ^2^ = 30.85, df = 3, *p* < 0.001) with their cats than those that self-identified as dog people. The majority (58.8%) of cat people engaged in active play with their cats for over 15 min, while only 44.7% of dog people actively played with their cat for more than 15 min. A majority (53.0%) of cat people also spent more than 6 h in passive interaction with their cats compared with only 34.9% of dog people (Table 5).

#### 3.4.2. Species Preferences and the Impact on Active and Passive Interactions in Dog-Owning Households

In dog-owning households, individuals who self-identified as dog people spent significantly more time engaging in both active play with their dogs (*n* = 616, χ^2^ = 24.46, df = 3, *p* < 0.001) and passive interaction with their dogs (*n* = 618, χ^2^ = 32.93, df = 3, *p* < 0.001) than those that self-identified as cat people. Less than 19% of cat people actively played with their dogs for more than 45 min. In comparison, over 35% of dog people played with their dogs for over 45 min per day. The majority (73.3%) of dog people spend more than 6 h in passive interactions with their dogs compared with half (51.7%) of cat people (Table 6).

#### 3.4.3. Species Preference and the Impact on Diets Fed in Cat-Owning Households

Pet preference yielded significant differences in the feeding of prescription and raw diets in cat-owning households. Cat people were significantly more likely to feed their cats a prescription diet for a medical condition as prescribed by their veterinarian compared with dog people (*n* = 574, χ^2^ = 16.53, df = 1, *p* < 0.001). Of the people who self-identified as cat people, 31.8% indicated that they were feeding their cats a veterinarian-prescribed diet for a medical condition compared with 17.2% of dog people (Table 7).

However, in cat-owning households, dog people were significantly more likely to feed their cats a raw diet compared with cat people (*n* = 574, χ^2^ = 6.32, df = 1, *p* = 0.012). Of those who self-identified as dog people, 6.3% indicated that they fed their cats a raw diet compared with 2.0% of cat people (Table 8). No other diet categories differed significantly between cat people and dog people in cat-owning households.

#### 3.4.4. Species Preferences and the Impact on Diets Fed in Dog-Owning Households

For the respondents that owned dogs, there was no significant difference noted in the number of cat people that fed their dogs a veterinarian-prescribed diet for a medical condition compared with the number of dog people that feed their dogs a veterinarian-prescribed diet for a medical condition (*n* = 620, χ^2^ = 0.44, df = 1, *p* = 0.506). Overall, 16.8% of dog owners fed their dogs prescription diets, regardless of identification as a cat person or a dog person (Table 9).

Conversely, in dog-owning households, individuals who self-identified as a dog person were significantly more likely to feed their dog a raw diet compared with those who self-identified as a cat person (*n* = 620, χ^2^ = 16.50, df = 1, *p* < 0.001). Only 2.8% of the cat people indicated that they fed their dogs a raw diet compared with 13.9% of the dog people (Table 10). No other diet categories differed significantly between cat people and dog people in dog-owning households.

## 4. Discussion

To our knowledge, this is the first study to investigate factors associated with the development of pet owners’ self-identified cat or dog preference and whether preferences correlated differentially with treatment of pets in single-species or multispecies pet (cat and dog) households. Some differences in demographics and exposure to cats or dogs when young were found between those who preferred cats or dogs as adults. We also found species preference to be correlated with several aspects of human–pet interactions and care of pets in the household.

### 4.1. Pet Ownership Demographics and Species Preferences

Findings confirmed our hypothesis that more people would consider themselves dog people (63.3%) than cat people (36.7%), and this aligns with previous pet preference surveys [24,25]. Dogs may have some intrinsic advantages to explain the apparent disparity in attractiveness among pet owners. Advanced interspecific human–dog communication in dogs seems to allow their social signaling to be more recognizable to humans [15,16,17]. While cats do exhibit human-specific communication, it has not been impacted by domestication to the extent seen in dogs [34,35], and studies have shown human interpretations of these signals are often inaccurate, such as misjudging the trill of a friendly feline greeting as anger [20]. Furthermore, when compared with cats, dogs seem to have more pronounced juvenile physical traits that selectively elicit caregiving and positive emotional responses in people, first described as Kinchenschema (baby schema) by Konrad Lorenz [10]. Research suggests that this phenomenon is found early in human development, as children as young as 3 to 6 years old were more attracted to canine than feline faces [36]. However, these preferences can be shaped over time by exposure to pets of specific species, the sex of the participant [36,37], and human personality traits [25]. Interestingly, a preference for cats over dogs was noted in both adults and children who had cats in their home when compared with those that did not, but a similar preference for dogs over cats was not found with dog ownership [36,37].

Cultural stereotypes about cats and dogs in the US may also factor into pet preference. According to a recent marketing survey, cats were often portrayed negatively in the media, especially when juxtaposed with a heroic canine character [38]. Cats as symbols of paganism and (female) witchcraft in Christian-dominated Europe during the Middle Ages continued to linger into early American superstitious folklore and literature [39] and underpin current stereotypes of “sad,” “spinster,” and “forever alone” female cat ownership in women [40,41,42]. Furthermore, feminine or less masculine attributes have been assigned to both men and women when associated with cats [23,24], and these stereotypes may influence pet preference and ownership.

Dwelling location was also significantly associated with pet preference. Cat people were more likely to live in an urban area (23.0% of cat people and 14.9% of dog people), whereas dog people tended to live in a rural area (26.1% of dog people and 16.7% of cat people), with suburban neighborhoods having an approximately equal distribution of either cat (60.3%) or dog people (59.0%). While this may simply reflect the perception that companion cats are more conducive to the space restrictions of urban dwellings, the role of pets in the choice of residence cannot be ruled out. For example, people strongly bonded to their dogs may opt for suburban or rural properties to more easily accommodate dogs’ exercise and enrichment needs. Underlying personality traits may influence an individual’s overall lifestyle, including both pet preference and living environment. Studies in geographical psychology looking at macrolevel perspectives have found that personality traits are geographically clustered [43,44,45], implying that it may be the individual’s personality that is driving their choice in living locale over their desire to provide the most ideal environment for their preferred pet species. Futures studies examining the interaction of personality, location, and pet preference may be helpful to better understand the human–animal bond.

It is not surprising that the self-identification of cat or dog preference affected the composition of pets in the household, in that cat people were more likely to have only cats in the home (29.6% of cat people vs. 0.9% of dog people) and dog people were more likely to live in dog-only homes (27.9% vs. 0.8% of cat people), but people in multispecies households with both cats and dogs were no more likely to identify as dog or cat people (69.6% of cat people and 71.2% of dog people). Cat people with cat-only households had a median of two cats per household, and dog people with dog-only households had a median of two dogs per household. This finding somewhat contradicts the stereotype that cat lovers commonly own large numbers of cats in cat-only households. We further found that in feline and canine multispecies homes, the cat person had a median of two cats and one dog, and a median of two dogs and one cat for dog people. This contrasts with the AVMA survey [1], which found the mean number of cats and dogs in the household to be similar (1.78 and 1.46, respectively). Dog owners in this survey may have more dogs than the average US household, and our participant population may be more willing to invest the effort and financial resources into multiple pets compared with the typical US pet owner.

### 4.2. The Development of Species Preferences—From Youth to Adulthood

Following the hypothesis that most people would prefer dogs irrespective of duration and at-home exposure because of the comparative ease in interpreting canine social signaling [15,16,17] and possible innate preference for dog faces [36], we proposed that individuals are more likely to identify as cat people only after extended contact with cats. For example, if one did not have a cat in the household as a child (before 18 years of age), one may be less likely to be a cat person when young, and more likely to change species preferences after exposure to cats as an adult.

First, we examined the stability of species preferences from youth to adulthood and found dog preference was more stable from childhood to adult (80.9%) compared with cat preference (54.5%). Species preference in dog people remains consistent from youth to adulthood, but adult cat people were equally likely to have been a cat or dog person while growing up, suggesting a higher chance of changing preferences from a dog to a cat later in life.

When specifically evaluating the groups that changed species preference from youth to adulthood, we found that 47.2% of Dog–Cat people (preference changed from dogs in childhood to cats in adulthood) grew up in dog-only homes, whereas only 24.4% of Cat–Dog people came from cat-only childhood homes. The lack of exposure to cats, but not dogs, in childhood was associated with future preference, in that individuals who changed from a dog to cat preference later in life were less likely to have access to cats in their childhood home. This provides some support for our hypothesis that exposure to cats in a home environment might be important to form stronger bonds and preference for cats. Furthermore, of the Dog–Cat people who did have cats when young, 50.0% were multispecies homes and almost none from cat-only homes (2.8%). As a mesopredator subject to predation by larger animals, cats may default to prey-like avoidance, hiding, and escape behaviors when they share a household with dogs. This in turn may result in an overall reduction in the time spent with humans in a cat and dog household. As such, the presence of dogs in a multispecies home is likely inhibitory to human–cat interactions, thereby further reducing the childhood cat exposure for Dog–Cat individuals.

It is worth noting that the other factors may also impact the quality of exposure. For example, those growing up with outdoor-only cats may be more likely to develop a preference for cats as an adult once residing with primarily indoor-only cats, or people who experienced an injury from a cat or dog when young may foster an aversion to that species. Other circumstances prompting a change, such as a partner or roommate bringing the animal into the same home or limitations to exposure due to a household member with a pet allergy, could also further elucidate the ontogeny of preference and the human–animal bond. Questions regarding some of these characteristics were queried and will be presented in future publications.

Finally, we found that people who changed species presences were more likely to live in a multispecies household as an adult, with no difference between the Cat–Dog and Dog–Cat groups. It appears that in our surveyed population, changing preference did not lead to abandoning the previously preferred species but rather introducing the newly preferred species into the household. Moreover, the finding that lack of exposure to cats when young might inhibit formation of an adult preference could present unique opportunities to address cat homelessness. Longer-term social efforts such as encouraging families with young individuals to foster cats or volunteer with cat rescues may result in species preference changes, ultimately creating more homes for cats in need.

### 4.3. Species Preferences and Interactions with Pets

Overall, almost half of pet owners reported actively playing with their cats and dogs for 15–45 min daily and spending more than 6 h per day in passive interaction with their pets. However, we found a correlation between an individual’s self-identified species preference and the amount of both active and passive interaction with the preferred and non-preferred species. In cat-owning households, cat people reported spending significantly more time in active play with their cats (58.8%, 15 min or more daily) compared with dog people (44.7%). Cat people were also more likely to spend more than 6 h within a few feet of their cat (53.0%) compared with dog people (34.9%). Similar preferred-species differences were seen with dogs, in that over 35% of dog people spent more than 45 min each day actively playing with their dogs compared with 19% of cat people. Almost three-fourths (73.3%) of dog people spend over 6 h passively interacting with their dogs versus 51.7% of cat people spending this amount of time in the vicinity of their dogs.

Play has been recommended as an important source of aerobic exercise and mental stimulation for pets and a method to induce positive affect for both the pet and human. Species-specific play is considered one of the Five Pillars of Feline Environmental Needs [46]. Increased interaction time both helps facilitate the formation of and serves as an indicator of a stronger human–animal bond [47,48,49]. Which party instigates the interaction may be especially relevant in cats, as previous studies have shown that in human–cat interactions, those initiated by cats were generally more successful and lasted longer than ones initiated by the human [49]. It appears that when it comes to human–feline interactions in particular, the cat plays as important of a role, if not more, in the quality of the interaction as the human; a quality which, in turn, may influence the formation of a species preference in the human. Interestingly, this is in direct contrast to human–dog interactions, in which dogs who initiated play more frequently were found to be significantly less amenable and more likely to display aggressive behaviors [50]. However, in general, activities involving physical contact and proximity during play have also been highly correlated with positive emotional scores in both dogs and their owners [51]. Therefore, the bias we found in active and passive interactions based on species preference could indicate suboptimal wellbeing for the less preferred species. These results are especially concerning given our finding that the majority (71.3%) of individuals that changed species preferences chose to live in multispecies homes. While people may choose to engage in more activities with their preferred species, another explanation might be related to conflict between the dogs and cats in a multispecies household, limiting the amount of time individuals of the two species spend near each other. The stronger fear response in cats may prompt more hiding and distance-increasing behaviors toward dogs, overall reducing the amount of time spent with humans when dogs are also present. The optimal amount of time playing or engaged in affiliative behaviors has yet to be determined; therefore, we cannot determine if the less preferred species in a multispecies household is indeed receiving a suboptimal amount of interaction with people in their household. Future studies should investigate the type of play (non-contact fetch or feather toy compared with tug or wrestling), who initiates the interaction, and the temperaments of the individual animals.

Pet owner personalities, emotional states, and perception of their pets have also been shown to affect interactions. Feline owners who perceived cats as family members were more likely to attribute positive traits like compassion, empathy, and loyalty to their cats [52], as well as provide their cats with more access to main areas of the house [53]. Some dog people, such as those with extroverted personalities, may gravitate towards the dogs in the home and be less educated about feline play styles of cats. For example, people only accustomed to the overt play solicitations from dogs may not be aware adult cats most commonly engage in object/predatory play with moving objects [54], and for indoor cats, this typically requires initial engagement from a person. There is also evidence that cats may initiate interactions with familiar people based on that person’s mood. Cats were shown to engage in more head and flank allorubbing with owners in a depressive mood compared with non-depressive owners [55] and approach owners that felt extroverted or agitated more often [56], suggesting that cats may possess a more sophisticated ability to read human emotions than what their domestication history implies. Additional studies regarding the interaction of the pet owner emotional state, the knowledge of basic needs for companion animal species, and pet preference could highlight pet owner populations in need of additional education.

### 4.4. Species Preferences and Pet Diet

Diet choice may be one indicator of the importance of health care to a pet owner and strength of the human–animal bond. Among the options in the current survey (commercial prescription diet prescribed by your veterinarian for a medical condition, a commercial diet from the pet store or a special pet food subscription service, a commercial diet from a grocery store/supermarket/warehouse store, a homecooked diet, and a raw diet), prescription and raw diets were found to significantly differ between cats and dogs, depending on the owner’s preferred species. There was no difference in pet owner’s species preference and prevalence of dogs fed prescription foods. However, in cat-owning households, cat people (31.8%) were significantly more likely to feed their cats a prescription diet compared with dog people (17.2%). The prevalence of chronic kidney disease (CKD) in cats is both high and increasing, with more recent studies suggesting that anywhere from 31% to 50% of cats are afflicted with this disease [57,58]. Feeding a renal prescription diet is an essential part of treating CKD and maintaining a good quality of life. As such, failing to provide medically indicated prescription diets may result in suboptimal health care and compromised welfare for a large percentage of cats. It is possible that fewer cats in dog people homes had medical conditions warranting a prescription diet or, due to fewer veterinary visits or early recognition of behavioral signs indicating illness, that dog people were less aware that their cats had a condition that could have benefited from specific dietary support. In addition to the increased cost, the inconvenience of separating prescription and non-prescription pet foods in a household with more than one pet or transitioning to a new and potentially less palatable prescription diet can all be barriers to choosing a prescription diet. Our findings indicate that cat people are more likely to endure the inconvenience and additional cost of a specialized diet for the health of their cats. Further studies should explore if preference extends to other aspects of pet health care, such has preventative care veterinary visits, more invasive surgical treatments, or specialized care, such as dermatological, behavioral, or oncological treatments, in the preferred and non-preferred species.

While relatively few participants in our study reportedly fed raw animal-based protein diets to their pets, dog people were more likely to feed both their dogs and cats raw diets compared with cat people. The American Veterinary Medical Association (AVMA) and the World Small Animal Veterinary Association (WSAVA) do not recommend the use of commercially available raw diets [59,60] due to evidence of various health and safety risks to both pets and humans [61] derived from pathogenic contamination [62,63,64] and potential nutritional imbalances associated with these diets [65,66,67]. Nevertheless, a small fraction of the veterinary medical community claim that there are benefits to feeding raw, such as the higher energy content and macronutrient digestibility of raw diets when fed to domestic cats [68] and potential benefits of feeding these diets to some exotic felids [69,70]. Previous studies of dog owners indicate that those who fed a raw diet perceived health benefits in their dogs and no health risks to themselves or dogs from raw diets but perceived an increased health risk from feeding commercial non-raw diets [71] and distrust of the commercial pet food industry [72,73]. The perceived benefits and low risks of raw diets seemed to appeal to more dog people in our study, and these beliefs are strong enough to extend to many of their cats. While controversial, the inconvenience and high cost of raw diets for pets in dog preferred households may be an indicator of a stronger human–animal bond in these homes.

### 4.5. Limitations

As with any survey study, the findings of this study may be influenced by the method of recruitment and not accurately reflect a wider population of cat and dog owners. The majority of respondents in our study lived with both cats and dogs (71%), followed by dog-only (18%) and cat-only households (11%). Multispecies pet homes were overrepresented in our participant population compared with the AVMA and APPA data from 2022, which showed 45% and 26% of US households with canine and feline companions, respectively, and 21% of households owning both cats and dogs [1,2]. These differences are likely reflective of specific sampling populations, although methodology details are not available for all studies. All the surveys used non-probability or non-random sampling techniques; thus, results may be limited to their specific sample populations. Our participants, having been recruited from veterinary hospitals and social media sites focused on veterinary interests, may have a different relationship and bond to their pets compared with those in other studies and in the broader population of pet owners. As such, our results likely represent highly motivated pet owners with strong interest in the health and behavior of their pets. If this is true, then the increased motivation of the pet owners we sampled may be impacting the magnitude of our findings; for instance, in a sample of pet owners with a lower interest in the health and behavior of their pets, the decreased interest of cat-owning dog people in feeding their cats prescription diets may be even more profound. Additional studies using more random sampling of the general pet population are needed.

We also limited the preference choices to dog or cat and did not provide an option for a preference for both species or neither species, nor did we query the age or gender of the survey participant. There is evidence that both additional choices and sex of the participant will influence results [25]. Since there is a paucity of data regarding the development and impact of pet preference to date, the goal of this exploratory study was to highlight a few initial significant findings from a more extensive survey. Future studies could provide more in-depth assessments of each area explored in this study. For example, existing attachment scales could be used as an indicator of preference (e.g., Lexington Attachment to Pets Scale [74], a partially validated pet attachment tool for both felines and canines). More nuanced questions regarding the quality of exposure to pets and barriers to ownership during childhood and as an adult are needed for examining the development of pet preferences. Additionally, important details about human–animal interactions and quality of pet care may be easy to overlook in surveys. Assessment of health care is complex, and we attempted to use diet choice, particularly prescription diets, as a proxy. However, a more in-depth, structured interview process could provide additional information about pets’ health and welfare, such as the pet’s body condition score and obesity, the caretaker’s willingness to seek medical care for various health concerns, and the assessment of owners’ level of awareness of chronic diseases and treatment options. The evaluation of factors within each of these areas is needed to better understand the interaction of individual experiences, temperaments, cultural influences, knowledge on quality of interactions, health care, and environment of pets in a household. The ultimate goal is identifying pets at risk of suboptimal wellbeing and increasing education for those pet owners.

## 5. Conclusions

This is the first study to assess lifestyle factors that correlate with both the development of pet preference and certain human–pet interactions at home. We found support for our hypotheses that more people would identify as dog people and that more exposure to cats (e.g., in a home environment) may be a factor in developing a cat preference compared with dogs and dog preference. For the individuals that changed species preferences from childhood to adulthood, we found that lack of childhood exposure to cats reduced the likelihood of developing a cat-species preference, but that was not noted for people who developed a preference for dogs. Owner’s species preferences were found to have wide-reaching associations with household pet-species demographics and treatment of the preferred and non-preferred species. Preferred species received more positive contact and specialized diets fed to pets. Pet preference was also correlated with dwelling population density, with cat people and dog people tending to live in urban and rural areas, respectively. Ultimately, more research is needed to fully understand the impact of species preferences on companion animal welfare in multispecies households.

## Figures and Tables

**Table 1:** Number and percentage of cat-only, dog-only, and multispecies (cat and dog) households based on species preferences.

	Cat Person	Dog Person	Total
Cats-only households	76 (29.6%)	4 (0.9%)	80 (11.4%)
Dog-only households	2 (0.8%)	124 (27.9%)	126 (18.0%)
Multispecies households	179 (69.6%)	316 (71.2%)	495 (70.6%)
Total households	257	444	701

**Table 2:** Number and percentage of participants with species preference when young and species preference as an adult.

	Adult Cat Person	Adult Dog Person	Total
Young Cat Person	140 (54.5%)	85 (19.1%)	225 (32.1%)
Young Dog Person	117 (45.5%)	359 (80.9%)	476 (67.9%)
Total	257	444	701

**Table 3:** Number and percentage of participants in the Cat–Dog and Dog–Cat groups who lived in single-species homes versus multispecies households when under the age of 18.

	Cat–Dog Person	Dog–Cat Person	Total
Cats only household when young	20 (24.4%)	3 (2.8%)	23 (12.2%)
Dogs only household when young	1 (1.2%)	50 (47.2%)	51 (27.1%)
Multispecies household when young	61 (74.4%)	53 (50.0%)	114 (60.6%)
Total	82	106	188

**Table 4:** Number of people in the Cat–Dog and Dog–Cat groups living in single-species homes versus multispecies households currently.

	Cat–Dog Person	Dog–Cat Person	Total
Cats only household currently	1 (1.2%)	35 (29.9%)	36 (1.78%)
Dogs only household currently	21 (24.7%)	1 (0.9%)	22 (10.9%)
Multispecies household currently	63 (74.1%)	81 (69.2%)	144 (71.3%)
Total	85	117	202

**Table 5:** In cat-owning households, the amount of time per day cat people and dog people spent in active play and passive interaction with cats.

		Cat Person	Dog Person	Total
Active play with cats (in minutes per 24-h day)	Less than 15 min	105 (41.2%)	177 (55.3%)	282 (49.0%)
	15–45 min	116 (45.5%)	107 (33.4%)	223 (38.8%)
	45–90 min	27 (10.6%)	30 (9.4%)	57 (9.9%)
	More than 90 min	7 (2.7%)	6 (1.9%)	13 (2.3%)
	Total	255	320	575
Passive interaction with cats (in hours per 24-h day)	Less than 3 h	39 (15.5%)	111 (34.9%)	150 (26.4%)
	3–5 h	79 (31.5%)	96 (30.2%)	175 (30.8%)
	6–8 h	69 (27.5%)	60 (18.9%)	129 (22.7%)
	More than 8 h	64 (25.5%)	51 (16.0%)	115 (20.2%)
	Total	251	318	569

**Table 6:** In dog-owning households, the amount of time per day cat people and dog people spent in active play and passive interaction with dogs.

		Cat Person	Dog Person	Total
Active play with dogs (in minutes per 24-h day)	Less than 15 min	59 (33.0%)	82 (18.8%)	141 (22.9%)
	15–45 min	87 (48.6%)	200 (45.8%)	287 (46.6%)
	45–90 min	26 (14.5%)	106 (24.3%)	132 (21.4%)
	More than 90 min	7 (3.9%)	49 (11.2%)	56 (9.1%)
	Total	179	437	616
Passive interaction with dogs (in hours per 24-h day)	Less than 3 h	27 (15.0%)	26 (5.9%)	53 (8.6%)
	3–5 h	60 (33.3%)	91 (20.8%)	151 (24.4%)
	6–8 h	39 (21.7%)	98 (22.4%)	137 (22.2%)
	More than 8 h	54 (30.0%)	223 (50.9%)	277 (44.8%)
	Total	180	438	618

**Table 7:** In cat-owning households, the number of cat people and dog people that fed their cats a veterinarian-prescribed diet for a medical condition.

	Cat Person	Dog Person	Total
Do NOT feed a prescription diet	174 (68.2%)	264 (82.8%)	438 (76.3%)
Feed a prescription diet	81 (31.8%)	55 (17.2%)	136 (23.7%)
Total	255	319	574

**Table 8:** In cat-owning households, the number of cat people versus dog people that fed their cats a raw diet.

	Cat Person	Dog Person	Total
Do NOT feed a raw diet	250 (98.0%)	299 (93.7%)	549 (95.6%)
Feed a raw diet	5 (2.0%)	20 (6.3%)	25 (4.4%)
Total	255	319	574

**Table 9:** In dog-owning households, the number of cat people and dog people that fed their dogs a veterinarian-prescribed diet for a medical condition.

	Cat Person	Dog Person	Total
Do NOT feed a prescription diet	147 (81.7%)	369 (83.9%)	516 (83.2%)
Feed a prescription diet	33 (18.3%)	71 (16.1%)	104 (16.8%)
Total	180	440	620

**Table 10:** In dog-owning households, the number of cat people and dog people that fed their dogs a raw diet.

	Cat Person	Dog Person	Total
Do NOT feed a raw diet	175 (97.2%)	379 (86.1%)	554 (89.4%)
Feed a raw diet	5 (2.8%)	61 (13.9%)	66 (10.6%)
Total	180	440	620

## Data Availability

The raw data supporting the conclusions of this article will be made available by the authors upon request.

## Supplementary Materials

The following supporting information can be downloaded at: https://www.mdpi.com/article/10.3390/ani14172534/s1, Table S1: Full questionnaire and informed consent form.
