# Supplementary material for: Evaluation of Characteristics Associated with Self-Identified Cat or Dog Preference in Pet Owners and Correlation of Preference with Pet Interactions and Care: An Exploratory Study
Source: Animals (Basel). 2024 Aug 31;14(17):2534. doi: 10.3390/ani14172534 (PMC11394057; doi:10.3390/ani14172534)
Supplement: Supplementary file 1 [file animals-14-02534-s001.zip › animals-3147957-supplementary.pdf]

Supplementary Table S1. Informed Consent and Survey Questions

Consent for Research Participation

Research Study Title: Human-animal interactions associated with self-identified cat or dog preference in pet owners.

Researcher(s):
- Dr. Julia Albright, University of Tennessee College of Veterinary Medicine
- Dr. Andrea Y. Tu, Heart of Chelsea Veterinary Group

We are asking you to be in this research study because you live in the United States and currently own a cat(s) a dog(s) OR both a cat(s) and a dog(s). You must be age 18 or older to participate in the study. The information in this consent form is to help you decide if you want to be in this research study. Please take your time reading this form and contact the researcher(s) to ask questions if there is anything you do not understand.

Why is the research being done?

The purpose of the research study is to look at the interactions of cats, dogs and humans living in the same household, and humans' perceptions of cats and dogs.
This study is being conducted by researchers at the University of Tennessee, Knoxville and researchers at Heart of Chelsea Veterinary Group.

What will I do in this study?

If you agree to be in this study, you will complete an online survey. The survey includes questions about your experiences living with cats or dogs as pets, and how your pets interact with you and each other at home. The survey should take you about 15 minutes to complete. You can skip questions that you do not want to answer.

Can I say "No"?

Being in this study is up to you. You can stop up until you submit the survey. After you submit the survey, we cannot remove your responses because we will not know which responses came from you. Either way, your decision won't affect your relationship with the University of Tennessee College of Veterinary Medicine, the Heart of Chelsea Veterinary Group or the services and health care you and your pet receive from your veterinarian.

Are there any risks to me?

We don't know of any risks to you from being in the study that are greater than the risks you encounter in everyday life. Thinking about pets can be emotional. If you feel you need to speak with someone about this, you may contact the University of Tennessee Veterinary Social Work service at 865-974-8839 or https://vetsocialwork.utk.edu.

Are there any benefits to me?

We do not expect you to benefit from being in this study. Your participation may help us to learn more about pet ownership. We hope the knowledge gained from this study will benefit others in the future.

What will happen with the information collected for this study?

The survey is anonymous, and no one will be able to link your responses back to you. Your responses to the survey will not be linked to your computer, email address or other electronic identifiers. Please do not include your name or other information that could be used to identify you in your survey responses. Information provided in this survey can only be kept as secure as any other online communication. Information collected for this study will be published and possibly presented at scientific meetings.

Will I be paid for being in this research study?

You will not be paid for being in this study.

Who can answer my questions about this research study?

If you have questions or concerns about this study or have experienced a research-related problem or injury, contact the researchers, Dr. Julia Albright (jalbrig1@utk.edu) or Dr. Andrea Y. Tu (dr.tu.dvm@gmail.com). For questions or concerns about your rights or to speak with someone other than the research team about the study, please contact:

Institutional Review Board
The University of Tennessee, Knoxville
1534 White Avenue
Blount Hall, Room 408
Knoxville, TN 37996-1529
Phone: 865-974-7697
Email: utkirb@utk.edu

Statement of Consent

I have read this form, been given the chance to ask questions and have my questions answered. If I have more questions, I have been told who to contact. By selecting "I Agree" below, I am providing my signature by electronic means and agree to be in this study. I can print or save a copy of this consent information for future reference. If I do not want to be in this study, I can select "I Do Not Agree" to exit out of the survey.

- I agree
- I do not agree

Do you live in the United States?

- Yes
- No

Are you 18 years of age or older?

- Yes
- No

Q1
How many cats and dogs in total are CURRENTLY living in your home?

- ○ Cats ____
- ○ Dogs ____

Q2
Please list the total number of **CATS CURRENTLY** living in your home ***in the order they entered your household***, starting from the most recently added cat. Note: there is only space for a total of 10 cats.

	Click to write Column 1
	Breed
Cat 1 Name	
Cat 2 Name	
Cat 3 Name	
Cat 4 Name	
Cat 5 Name	
Cat 6 Name	
Cat 7 Name	
Cat 8 Name	
Cat 9 Name	
Cat 10 Name	

Q3
Please list where you obtained each **CAT**.
[Dropdown menu options are:
- *Breeder*
- *Shelter/Rescue*
- *Stray*
- *Private/Direct Adoption from Previous Owner (Not from professional breeder or shelter group)*
- *Other]*

	Where obtained	If Other is selected
		please describe
Cat 1 Name	▼ *Dropdown menu*	
Cat 2 Name	▼ *Dropdown menu*	
Cat 3 Name	▼ *Dropdown menu*	
Cat 4 Name	▼ *Dropdown menu*	
Cat 5 Name	▼ *Dropdown menu*	
Cat 6 Name	▼ *Dropdown menu*	
Cat 7 Name	▼ *Dropdown menu*	
Cat 8 Name	▼ *Dropdown menu*	
Cat 9 Name	▼ *Dropdown menu*	
Cat 10 Name	▼ *Dropdown menu*	

Q4

Do any of your **CATS** show these behaviors or have they been diagnosed by a veterinarian with one of the following (choose all that apply):

- Hiss/growl or swat at people more than once a week
- Hiss/growl or swat at other cats in the household more than once a week
- Hiss/growl or swat at dogs in the household more than once a week
- Overgrooming (hairless areas)
- Urinate or defecate outside the litterbox more than once a month
- Recurrent urinary tract disease (Feline Idiopathic cystitis or urinary tract infection (UTI))
- Vomiting multiple times a month
- Choosing to spend time in a protected/separate space away from people in the household, e.g. alone under the bed.
- Choosing to spend time in a protected/separate space away from other cats in the household, e.g. alone under the bed.
- Choosing to spend time in a protected/separate space away from dogs in the household, e.g. alone under the bed.

Q5

What type of diet do you feed your **CAT(S)** (Select all that applies):

- A commercial diet prescribed by your veterinarian for a medical condition (e.g., renal disease)
- A commercial diet from the pet store or a special petfood subscription service
- A commercial diet from a big box store (e.g. Costco) or a grocery store/supermarket
- Homecooked diet
- Raw diet
- Other

Q6
Please list the total number of **DOGS CURRENTLY** living in your home ***in the order they entered your household***, starting from the most recently added dog. Note: there is only space for a total of 10 dogs.

	Click to write Column 1
	Breed
Dog 1 Name	
Dog 2 Name	
Dog 3 Name	
Dog 4 Name	
Dog 5 Name	
Dog 6 Name	
Dog 7 Name	
Dog 8 Name	
Dog 9 Name	
Dog 10 Name	

Q7
Please list where you obtained each **DOG**.
[Dropdown menu options are:

- *Breeder*
- *Shelter/Rescue*
- *Stray*
- *Private/Direct Adoption from Previous Owner (Not from professional breeder or shelter group)*
- *Other]*

	Where obtained	If Other is selected
		please describe.
Dog 1 Name	▼ *Dropdown menu*	
Dog 2 Name	▼ *Dropdown menu*	
Dog 3 Name	▼ *Dropdown menu*	
Dog 4 Name	▼ *Dropdown menu*	
Dog 5 Name	▼ *Dropdown menu*	
Dog 6 Name	▼ *Dropdown menu*	
Dog 7 Name	▼ *Dropdown menu*	
Dog 8 Name	▼ *Dropdown menu*	
Dog 9 Name	▼ *Dropdown menu*	
Dog 10 Name	▼ *Dropdown menu*	

Q8

Do any of your **DOGS** show these behaviors or have been diagnosed by a veterinarian with one of the following (choose all that apply):

- Growl or snap/bite at people more than once a week
- Growl or snap/bite at other dogs in the household more than once a week
- Growl or snap/bite at cats in the household more than once a week
- Overgrooming (hairless areas) or excessive licking of paws/body parts
- Urinate or defecate in the house (aka house soil) more than once a month
- Diarrhea multiple times a month
- Choosing to spend time in a protected/separate space away from people in the household, e.g. alone under the bed.
- Choosing to spend time in a protected/separate space away from other dogs in the household, e.g. alone under the bed.
- Choosing to spend time in a protected/separate space away from cats in the household, e.g. alone under the bed.

Q9

What type of diet do you feed your **DOG(S)** (Select all that applies):

- A commercial diet prescribed by your veterinarian
- A commercial diet from the pet store or a special petfood subscription service
- A commercial diet from a big box store (e.g. Costco) or a grocery store/supermarket
- Homecooked diet
- Raw diet
- Other

Q10
Does the household consist of (check all that apply):

- ☐ Roommates
- ☐ Married couple/Domestic partnership
- ☐ Children under the age of 18
- ☐ Other family (e.g. adult parents)

Q11
Is your household located in a:

- ○ Rural area
- ○ Suburban area
- ○ Urban area

Q12
Number of adults in the household:

____

Q13
Number of people under the age of 18 currently living in the household:

____

This next set of questions will ask you about your experiences with cats and dogs when you were YOUNG/GROWING UP (less than 18 years of age).

Q14
What type of "person" would you consider yourself when **YOUNG** (less than 18 years old)?

- ○ Cat person
- ○ Dog person

This next set of questions will ask you about your experiences with cats and dogs now, as an ADULT (over 18 years of age).

Q15
What type of "person" would you consider yourself now (as an **ADULT**)?

- ○ Cat person
- ○ Dog person

Q16
Please let us know if you have any additional comments regarding your cat or dog preferences.

____

____

____

____

This final section asks you about how you, members of your household, your cat(s) and your dog(s) interact with each other in your home.

Q17:
On average, about how much time each day do **YOU** spend actively playing with your **CAT(s)** (e.g., playing fetch, using a fishing rod toy, etc.)? Please answer in minutes per 24-hour day.

- ○ None
- ○ Less than 15 minutes per day
- ○ 15-30 minutes per day
- ○ 30-45 minutes per day
- ○ 45-60 minutes per day
- ○ 60-90 minutes per day
- ○ 90-120 minutes per day
- ○ More than 120 minutes per day

Q18:
On average, about how much time each day do **PEOPLE IN THE HOUSEHOLD (including you)** spend actively playing with your **CAT(s)** (e.g., playing fetch, using a fishing rod toy, etc.)? Please answer in minutes per 24-hour day.

- None
- Less than 15 minutes per day
- 15-30 minutes per day
- 30-45 minutes per day
- 45-60 minutes per day
- 60-90 minutes per day
- 90-120 minutes per day
- More than 120 minutes per day

Q19:
On average, about how much time each day **YOU** spend actively playing with your **DOG(s),** (e.g., playing fetch, tug, or wrestling, etc.; do not include walks and runs unless you play these games during the outing)? Please answer in minutes per 24-hour day.

- None
- Less than 15 minutes per day
- 15-30 minutes per day
- 30-45 minutes per day
- 45-60 minutes per day
- 60-90 minutes per day
- 90-120 minutes per day
- More than 120 minutes per day

Q20:
On average, about how much time each day do **PEOPLE IN THE HOUSEHOLD (including you)** spend actively playing with your **DOG(s),** (e.g., playing fetch, tug, or wrestling, etc.; do not include walks and runs unless you play these games during the outing)? Please answer in minutes per 24-hour day.

- ○ None
- ○ Less than 15 minutes per day
- ○ 15-30 minutes per day
- ○ 30-45 minutes per day
- ○ 45-60 minutes per day
- ○ 60-90 minutes per day
- ○ 90-120 minutes per day
- ○ More than 120 minutes per day

Q21:
On average, about how much time each day do **YOU** spend in a passive state with your **CAT(s),** -e.g., the cat is sitting/lying on or within a few feet of a human, but not actively playing with the human(s)? Please answer in hours per 24-hour day.

- ○ None
- ○ Less than an hour per day
- ○ 1-2 hours per day
- ○ 3-5 hours per day
- ○ 6-8 hours per day
- ○ More than 8 hours per day

Q22:
On average, about how much time each day do **PEOPLE IN THE HOUSEHOLD (including you)** spend in a passive state with your **CAT(s),** - e.g., the cat is sitting/lying on or within a few feet of a human, but not actively playing with the human(s)? Please answer in hours per 24-hour day.

- ◯ None
- ◯ Less than an hour per day
- ◯ 1-2 hours per day
- ◯ 3-5 hours per day
- ◯ 6-8 hours per day
- ◯ More than 8 hours per day

Q23:
On average, about how much time each day do **YOU** spend in a passive state with your **DOG(s),** - e.g. the dog is sitting/lying on or within a few feet of a human but not actively playing with the human(s)? Please answer in hours per 24-hour day.

- ◯ None
- ◯ Less than an hour per day
- ◯ 1-2 hours per day
- ◯ 3-5 hours per day
- ◯ 6-8 hours per day
- ◯ More than 8 hours per day

Q24:
On average, about how much time each day do **PEOPLE IN THE HOUSEHOLD (including you)** spend in a passive state with your **DOG(s),** - e.g. the dog is sitting/lying on or within a few feet of a human, but not actively playing with the human(s)? Please answer in hours per 24-hour day.

- ○ None
- ○ Less than an hour per day
- ○ 1-2 hours per day
- ○ 3-5 hours per day
- ○ 6-8 hours per day
- ○ More than 8 hours per day
